# Editorial: Novel Technology in Psychiatric Rehabilitation

**DOI:** 10.3389/fpsyt.2022.979888

**Published:** 2022-07-18

**Authors:** Hector W. H. Tsang, Jessie J. Lin

**Affiliations:** ^1^Department of Rehabilitation Sciences, The Hong Kong Polytechnic University, Kowloon, Hong Kong SAR, China; ^2^Mental Health Research Centre, The Hong Kong Polytechnic University, Kowloon, Hong Kong SAR, China

**Keywords:** digital mental health, psychiatric rehabilitation, attitudes and knowledge, virtual reality, technology-ICT

Psychiatric rehabilitation aims to help people with mental disorders work toward and achieve personal and functional goals. Technology has now become an important part of rehabilitation strategies. New technologies provide unlimited possibilities to help foster participation and to create opportunities that were previously not possible. The ongoing global COVID-19 pandemic has driven the implementation and development of assistive technologies for mental health, such as web-based assessment and intervention, mobile applications, and virtual reality (VR). There have been a number of studies investigating the delivery mode of digital mental health services and the influencing factors related to their usage and implementation (1). Other studies have also investigated the feasibility and effectiveness of digital mental health interventions on various mental disorders (2–4). The digital revolution is rapidly transforming mental health care and psychiatric rehabilitation services, with a focus on big data, computing power, network-based information, and mobile and virtual technologies.

Mental disorders cause substantial health and societal burdens worldwide (5). A recent WHO-led study estimated that depression and anxiety disorders cost approximately US$1 trillion in lost productivity each year. Digital technologies have great potential for the assessment and delivery of interventions. Moreover, technology can enhance the reach of mental health care services due more convenient access and lower costs, particularly for those on low income or the older population. The five articles selected in this issue illustrate the potential of digital therapeutics for mental health, including a conventional intervention for ameliorating stress and anxiety in children with special needs using novel measures, a discussion on the attitudes and knowledge of different stakeholders on the use of advanced VR technologies in psychiatric care, and a study highlighting the barriers and facilitators to technology use in psychiatric rehabilitation. We also explore how to integrate digital technology in clinical practice to deliver high-quality, efficient, and timely mental health care, with an emphasis on individual centralization.

“Technology” is defined as a tool, means of modification, or a technique used to solve a problem, improve an existing intervention, or investigate the underlying mechanisms of a specific treatment. Educational kinesiology is a popular intervention in schools that uses physical movement to improve brain function. However, lack of scientific evidence supporting the effectiveness of some interventions has led to claims of pseudoscience. Tai et al. conducted a study with a quasi-experimental design and novel measures to examine the effect of educational kinesiology on salivary cortisol and oxytocin levels in 37 kindergarten children with special needs. The intervention group demonstrated significantly increased oxytocin levels compared to the waitlist control group, indicating a biophysiological pathway via elevated oxytocin levels that contributes to the anti-anxiety effects of educational kinesiology. The promising and encouraging findings in this study set the stage for further research to better understand the underlying mechanisms of such interventions and to facilitate their implementation in school settings.

Digital technologies are now playing a key role in mental health service reform worldwide. Digital innovation within health care services largely depends on people (i.e., clinicians, therapists, and service users) to achieve a value-driven, effective, and sustainable transformation. Thus, a comprehensive understanding of the attitudes and perceptions of frontline staff is needed. Gemesi et al. conducted an online survey of nutritionists to collect data on their use of behavioral and body image therapies, and attitudes toward the use of VR technology for treating obesity. The survey showed that most of the nutritionists used conventional behavioral therapy (96%) for treating obesity patients, whereas body image therapy (66%) was used less. Almost all participants (99%) reported they had no experience of using VR technology in their daily practice. However, the participants demonstrated a positive attitude toward the use of innovative technologies for obesity.

Chung et al. conducted semi-structured qualitative interviews in a group of cross-disciplinary clinicians and service managers in a major private mental health hospital in Australia. The study explored the perspectives of key stakeholders on the implementation of therapeutic VR technology in psychiatric care. They identified three major themes regarding the application of VR technology in mental health service settings: clinical factors, organizational factors, and professional factors. Each theme included both enablers and barriers that need to be addressed in order to successfully implement the use of VR technology in mental health care. The clinical factors emphasized the knowledge/perceptions for appropriate clinical implementation, including therapeutic effectiveness, safety, ethical issues, and patient engagement. The organizational factors captured the importance of service contexts, including cost, manpower, and logistical resourcing challenges. The professional factors covered the perceived acceptability and feasibility of its application at the manpower level, including the impact of staff attitudes toward the technology and perceived usability of the technology, which highlighted the need for staff education and training. Based on the results of the qualitative interviews, Chung et al. administered a more detailed online survey to explore the attitudes of 81 frontline personnel from a network of private psychiatric hospitals. The results showed that 91% of participants reported they had knowledge of VR technology, but only half of them were aware of its use in a mental health clinical setting. Most participants considered VR technology to be acceptable (84%), appropriate as a mental health treatment (69%), and feasible to be introduced into mental health services (59%).

Digital platforms provide a unique opportunity for the use of digital technologies in mental health care, which hold promise to enhance the reach and delivery of services and treatments. The above findings show that frontline personnel have a positive attitude toward the implementation of VR treatments for mental health, but few are actually familiar with the technologies. The future dissemination and implementation of digital technologies in mental health should focus on addressing the knowledge and skill gaps of professionals by providing training programs and development resources. The development of evidence-based practice guidelines is also needed to regulate the ethical and safe usage of therapeutic VR technology.

Beyond VR technology, advances in mobile communication and artificial intelligence (AI) offer great potential for enhancing mental health care by providing up-to-date “real-time” information. Ecological Momentary Assessment (EMA) and Ecological Momentary Intervention (EMI) are methods for repeated sampling of momentary data, which can facilitate multiple time-varying interventions over a short period in ecological contexts. Prof. Hector Tsang and his team have been investigating the feasibility of using EMA techniques in a mobile application to identify children at risk of depression and anxiety. The aim is to examine the effects of psychosocial interventions delivered via a mobile application on the physical and psychological wellbeing of people with mental disorders across different age groups. The use of EMA and EMI can extend and enhance the quantity and quality of mental health data from traditional provider-delivered health care to enabling remote and real-time data collection and delivery of interventions. Integrating EMA and EMI with AI also brings many opportunities for the application of digital technologies in personalized mental health care.

## Conclusion

This issue presents up-to-date knowledge and trends on the use of new technologies in psychiatric rehabilitation. The articles in this special issue describe the attitudes and knowledge on the implementation of technology across different stakeholders, and highlights the feasibility and necessity of the application of digital technologies in mental health care. Frontline professionals and staff must be heavily involved in designing and implementing technologies to successfully realize a digital mental health care service. Dissemination of these technologies should focus on understanding the attitudes and concerns of professionals to identify the barriers and enablers, ensuring service providers are well equipped with relevant knowledge and skills, and making sure that the service context is appropriate and feasible to aid adoption and sustained uptake of digital technologies. However, more studies are needed to provide evidence for digital interventions in a wide variety of mental disorders for clinical practice. Moreover, the underlying neurophysiological mechanisms of digital interventions are still unknown. Cross-disciplinary collaboration and evidence-based practice guidelines are also needed for safe and ethical use of therapeutic digital technologies. We hope this editorial will encourage rigorous studies to investigate the development and implementation of various innovative digital technologies for psychiatric rehabilitation, taking into account both the complexity of clinical practice and the added value of digital technology in the field.

## Author contributions

HT conceived and designed the idea, contributed to the writing, and editing. JL contributed to the manuscript writing and reference searching. Both authors contributed to the article and approved the submitted version.

## Conflict of interest

The authors declare that the research was conducted in the absence of any commercial or financial relationships that could be construed as a potential conflict of interest.

## Publisher's note

All claims expressed in this article are solely those of the authors and do not necessarily represent those of their affiliated organizations, or those of the publisher, the editors and the reviewers. Any product that may be evaluated in this article, or claim that may be made by its manufacturer, is not guaranteed or endorsed by the publisher.
